# Independent associations of *TOMM40* and *APOE* variants with body mass index

**DOI:** 10.1111/acel.12869

**Published:** 2018-11-21

**Authors:** Alexander M. Kulminski, Yury Loika, Irina Culminskaya, Jian Huang, Konstantin G. Arbeev, Olivia Bagley, Mary F. Feitosa, Joseph M. Zmuda, Kaare Christensen, Anatoliy I. Yashin, Long Life Family Study Research Group

**Affiliations:** ^1^ Biodemography of Aging Research Unit, Social Science Research Institute Duke University Durham North California; ^2^ Division of Statistical Genomics, Department of Genetics Washington University School of Medicine St Louis Missouri; ^3^ Department of Epidemiology, Graduate School of Public Health University of Pittsburgh Pittsburgh Pennsylvania; ^4^ The Danish Aging Research Center University of Southern Denmark Odense C Denmark

**Keywords:** age‐dependent effect, aging, *ApoE* polymorphism, body mass index, health span, lifespan, *TOMM40*

## Abstract

The *TOMM40‐APOE* variants are known for their strong, antagonistic associations with Alzheimer's disease and body weight. While a stronger role of the *APOE* than *TOMM40* variants in Alzheimer's disease was suggested, comparative contribution of the *TOMM40‐APOE* variants in the regulation of body weight remains elusive. We examined additive effects of rs2075650 and rs157580 *TOMM40* variants and rs429358 and rs7412 *APOE* variants coding the ε2/ε3/ε4 polymorphism on body mass index (BMI) in age‐aggregated and age‐stratified cohort‐specific and cohort‐pooled analysis of 27,863 Caucasians aged 20–100 years from seven longitudinal studies. Minor alleles of rs2075650, rs429358, and rs7412 were individually associated with BMI (*β *= −1.29, *p* = 3.97 × 10^−9^; *β *= −1.38, *p* = 2.78 × 10^−10^; and *β *= 0.58, *p* = 3.04 × 10^−2^, respectively). Conditional analysis with rs2075650 and rs429358 identified independent BMI‐lowering associations for minor alleles (*β *= −0.63, *p* = 3.99 × 10^−2^ and *β *= −0.94, *p* = 2.17 × 10^−3^, respectively). Polygenic mega‐analysis identified additive effects of the rs2075650 and rs429358 heterozygotes (*β *= −1.68, *p* = 3.00 × 10^−9^), and the strongest BMI‐lowering association for the rs2075650 heterozygous and rs429358 minor allele homozygous carriers (*β *= −4.11, *p* = 2.78 × 10^−3^). Conditional analysis with four polymorphisms identified independent BMI‐lowering (rs2075650, rs157580, and rs429358) and BMI‐increasing (rs7412) associations of heterozygous genotypes with BMI. Age‐stratified conditional analysis revealed well‐powered support for a differential and independent association of the rs429358 heterozygote with BMI in younger and older individuals, *β *= 0.58, 95% confidence interval (CI) = −1.18, 2.35, *p* = 5.18 × 10^−1^ for 3,068 individuals aged ≤30 years and *β *= −4.28, CI = −5.65, −2.92, *p* = 7.71 × 10^−10^ for 6,052 individuals aged >80 years. *TOMM40* and *APOE* variants are independently and additively associated with BMI. The *APOE* ε4‐coding rs429358 polymorphism is associated with BMI in older individuals but not in younger individuals.

## 1 INTRODUCTION

Studies report that deviation from the normal body weight is associated with cognitive decline and development of dementia and Alzheimer's disease (AD) (Emmerzaal, Kiliaan, & Gustafson, 2015). Despite these results are challenged in Fitzpatrick et al. (2009), they suggest that there might be common underlying biological mechanisms involved in regulation of body weight and cognitive function and/or AD pathology (Hinney et al., 2014). The relationship between body weight and cognitive function is complex as it may vary with age from a positive relationship between mid‐life obesity and AD to a negative relationship in late life (Emmerzaal et al., 2015).

High throughput genotyping of large human samples provides an opportunity for designing well‐powered studies to examine whether the same genetic variants can be associated with body weight and AD. The 19q13.3 genomic region harboring the *APOE* (apolipoprotein E) and *TOMM40* (translocase of outer mitochondrial membrane 40 homolog) genes represents an opportunity for such an analysis as it harbors pleiotropic variants conferring the strongest, well‐documented risk of AD and robust associations with BMI (body mass index, kg/m^2^) (Guo et al., 2013; Roses et al., 2010). Indeed, this locus harbors common *APOE* ε2/ε3/ε4 polymorphism, coded by rs429358 and rs7412 SNPs (single nucleotide polymorphisms). The *APOE* ε4 allele has been most consistently associated with AD in late life in various populations (Raichlen & Alexander, 2014). Genome‐wide association studies (GWAS) also report an AD‐increasing effect of the rs2075650 (*TOMM40*) minor allele and AD‐decreasing effect of the rs157580 (*TOMM40*) minor allele (Bao, Wang, & Mao, 2016; Harold et al., 2009). The association of rs2075650 with AD is often attributed (Yu et al., 2007) to linkage disequilibrium (LD) of this SNP with rs429358, which encodes the *APOE* ε4 allele.

Large‐scale meta‐analysis of populations of European ancestry reported a genome‐wide significant association of rs2075650 with BMI, with the AD‐risk‐increasing (minor) allele associated with smaller BMI (Guo et al., 2013). Whether the effect of this SNP should be attributed to *TOMM40* or *APOE* and, particularly, to the ε4 allele, has not yet been addressed in large samples (Guo et al., 2013). Thus, we performed cohort‐specific and cohort‐pooled analysis of 27,863 participants from seven independent longitudinal studies to comprehensively examine the associations of rs2075650 and rs157580 *TOMM40* variants and rs429358 and rs7412 variants coding the *APOE* ε2/ε3/ε4 polymorphism with BMI in a Caucasian ancestry population sample as well as in subpopulations of younger and older individuals.

## 2 RESULTS

Data on 27,863 participants were obtained from seven longitudinal cohort studies (ARIC, CHS, CARDIA, MESA, HRS, LLFS, and FHS) (see Section 4). Baseline measurements of BMI, the number of longitudinal measurements of BMI used in the analysis, and basic demographic information for the genotyped participants in each cohort and the pooled sample are presented in Supporting Information Table S1). Frequency distributions of genotypes for rs2075650, rs157580, rs429358, rs7412, and *APOE* ε2/ε3/ε4 (coded by rs429358 and rs7412, Supporting Information Table S2) polymorphisms are presented in Supporting Information Table S3. All polymorphisms were in Hardy–Weinberg equilibrium.

### 2.1 LD structure

**Figure 1:**
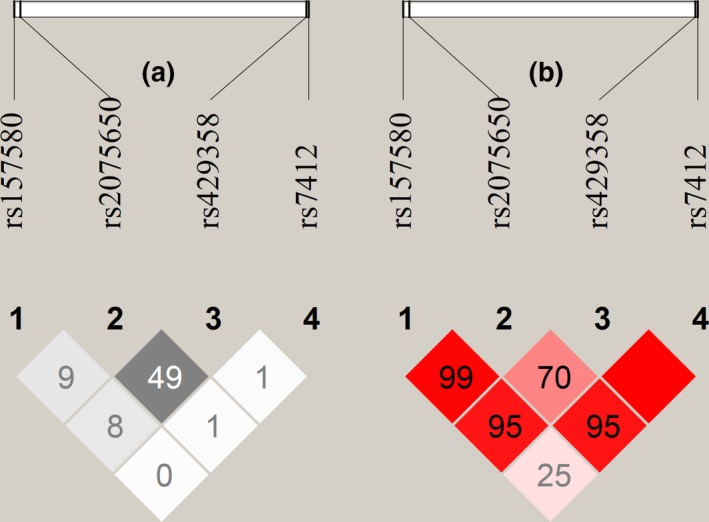
LD pattern of four SNPs in the *TOMM40–APOE* locus. LD (%), *r* ^2^ (a) and D' (b), is shown in the pooled sample of all cohorts

The largest LD (*r* ^2^ = 49%) was observed between rs429358 and rs2075650 (Figure 1) and was due to clustering of minor alleles of these SNPs rather than their minor and major alleles (Supporting Information Tables S4A and S4G). In contrast, LD between rs157580 and rs429358 was more modest (*r* ^2^ = 8%) and due to depleted clustering of minor alleles of these SNPs and preferable clustering of their minor and major alleles (Supporting Information Tables S4B and S4H). For the other SNPs pairs, LD measured by *r* ^2^ was small. Lewontin's D' varied from 25% to 100% (Figure 1).

### 2.2 Univariate associations

**Figure 2:**
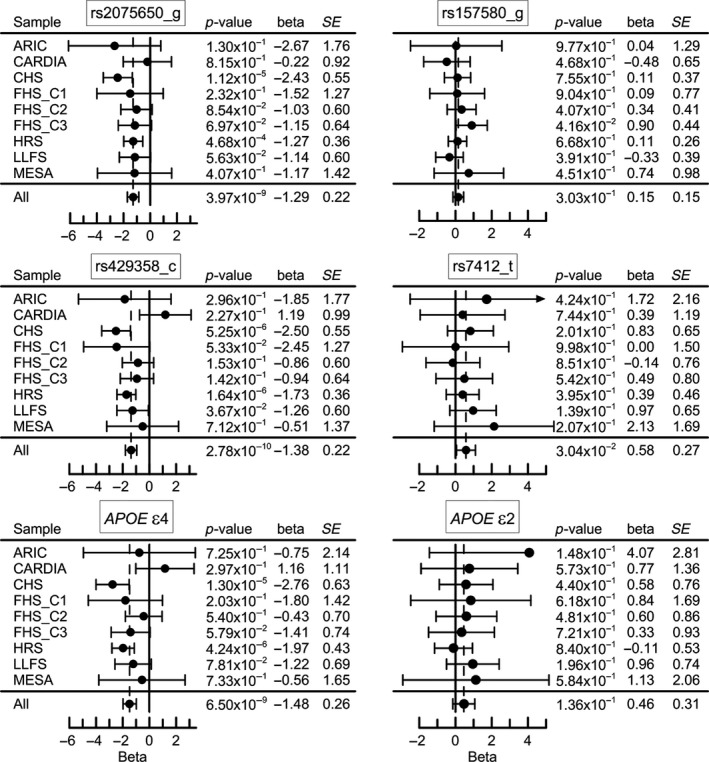
Genetic associations with BMI in each cohort and in the pooled sample of all cohorts (all). Associations of rs2075650 and rs157580 polymorphisms are from additive genetic model with minor allele as an effect allele. Statistical models for *APOE* were fitted considering the effect of the ε2 and ε4 alleles as compared with the ε3ε3 reference genotype. *SE* denotes standard error. Horizontal bars indicate 95% confidence intervals

In univariate analyses of single polymorphisms in the models, the rs2075650 minor allele was associated with decreased BMI in each cohort that resulted in a genome‐wide significant association in the pooled sample of all cohorts (*β *= −1.29, *p* = 3.97 × 10^−9^; Figure 2). The rs429358 minor allele, coding the *APOE* ε4 allele, showed the same‐direction genome‐wide significant effect in this sample (*β *= −1.38, *p* = 2.78 × 10^−10^). The rs7412 minor allele, coding the *APOE* ε2 allele, showed the opposite‐direction nominally significant effect in mega‐analysis (*β* = 0.58, *p* = 3.04 × 10^−2^). Mega‐analysis did not show significant association for rs157580 (*β* = 0.15, *p* = 0.303). The associations for the *APOE* ε2 allele, defined as the ε2ε2 or ε2ε3 genotypes, and the ε4 allele, defined as the ε3ε4 or ε4ε4 genotypes, resembled those for rs7412 and rs429358, respectively, except the association of ε2 allele with BMI was non‐significant (*β* = 0.46, *p* = 0.136). Mega‐analysis of the effects of each *APOE* genotype compared with the ε3ε3 genotype showed highly significant associations for the ε3ε4 (*β *= −1.41, *p* = 7.68 × 10^−8^) and ε4ε4 (*β *= −2.41, *p* = 3.32 × 10^−3^) genotypes and suggestive‐effect significance for the ε2ε3 genotype (*β *= 0.55, *p* = 8.26 × 10^−2^) (Table 1, Model 1).

**Table 1:** Univariate and multivariate associations of selected polymorphisms with BMI in a mega sample of 27,863 individuals from seven longitudinal studies

Polymorphism	Model 1			Model 2			Model 3			Model 4			Model 5		
	*β*	*SE*	*p*‐value	*β*	*SE*	*p*‐value	*β*	*SE*	*p*‐value	*β*	*SE*	*p*‐value	*β*	*SE*	*p*‐value
rs2075650^a^	−1.29	0.22	3.97E‐09	−0.63	0.31	3.99E‐02	−1.25	0.22	1.36E‐08	−0.61	0.31	4.71E‐02	−0.67	0.30	2.63E‐02
rs157580^a^	0.15	0.15	3.03E‐01												
rs429358^a^	−1.38	0.22	2.78E‐10	−0.94	0.31	2.17E‐03									
rs7412^a^	0.58	0.27	3.04E‐02				0.41	0.27	1.30E‐01						
ε2ε2^b^	−1.23	1.30	3.45E‐01							−1.28	1.30	3.27E‐01			
ε2ε3^b^	0.55	0.31	8.26E‐02							0.52	0.32	9.81E‐02			
ε2ε4^b^	−0.66	0.71	3.54E‐01							−0.27	0.74	7.20E‐01			
ε3ε4^b^	−1.41	0.26	7.68E‐08							−0.99	0.34	3.48E‐03			
ε4ε4^b^	−2.41	0.82	3.32E‐03							−1.56	0.92	9.26E‐02			
ε2^c^	0.46	0.31	1.36E‐01										0.43	0.31	1.63E‐01
ε4^c^	−1.48	0.26	6.50E‐09										−0.98	0.34	3.97E‐03

Notes

Model 1: Associations of rs2075650, rs157580, rs429358, rs7412, *APOE* genotypes, and *APOE* alleles separately. The *APOE* ε2 allele was defined as the ε2ε2 or ε2ε3 genotypes. The *APOE* ε4 allele was defined as the ε3ε4 or ε4ε4 genotypes. The ε2/ε4 genotype was excluded from definition of the ε2 or ε4 carrier status.

Model 2: Bivariate model of additive effects of rs2075650 and rs429358 SNPs.

Model 3: Bivariate model of additive effects of rs2075650 and rs7412 SNPs.

Model 4: Multivariate model of additive effects of rs2075650 and *APOE* genotypes.

Model 5: Multivariate model of additive effects of rs2075650 and *APOE* alleles.

a Additive genetic model with minor allele as an effect allele.

b Genotypic model for *APOE* with the ε3ε3 genotype as a reference.

c Allelic model for *APOE* with the ε3ε3 genotype as a reference.

### 2.3 Independent associations of *TOMM40* and *APOE* polymorphisms with BMI

Despite moderate LD between rs2075650 and rs429358 (Figure 1), bivariate mega‐analysis with these SNPs identified their independent associations with BMI, although their effects became smaller (Table 1, Model 2). Bivariate mega‐analysis with rs2075650 and rs7412 showed minor role of rs7412 in the association of rs2075650 with BMI (Table 1, Model 3). Conditional mega‐analysis with rs2075650 and *APOE* genotypes (Table 1, Model 4) or ε2 and ε4 alleles (Table 1, Model 5) resembled the results of the bivariate analysis. Association of rs2075650 with BMI, independent of the ε4 allele, was confirmed in the mega‐analysis of carriers of the ε3ε3 genotype only (*β *= −1.01, *p* = 2.65 × 10^−2^; Supporting Information Table S5).

Rs157580 did not attenuate the association of rs2075650, rs429358, rs7412, or ε2/ε3/ε4 polymorphisms with BMI (Supporting Information Table S6). The effect of rs157580 remained non‐significant but its direction changed aligning with the effect of the ε4 allele in all models except that with rs7412 (Supporting Information Table S6, Model 4).

Conditional mega‐analysis, with rs2075650, rs157580, rs429358, and rs7412 in the model, showed that all heterozygotes were independently associated with BMI (Table 2). Heterozygotes of rs2075650, rs429358, and rs157580 showed BMI‐lowering associations, whereas the rs7412 heterozygote (coding the *APOE* ε2ε3 and ε2ε4 genotypes) showed BMI‐increasing association. Conditional mega‐analysis with rs2075650, rs157580, and *APOE* genotypes did not show significant effects for either the ε2ε3 (*β* = 0.53, *p* = 9.25 × 10^−2^) or ε2ε4 (*β *= −0.11, *p* = 8.86 × 10^−1^) genotypes alone (Supporting Information Table S7) implying that examination of rs429358 and rs7412 SNPs rather than the *APOE* genotypes can help in separating the effects of the ε2 and ε4 alleles. The rs429358 minor allele homozygote (coding the *APOE* ε4ε4 genotype) was significantly associated with smaller BMI, whereas the BMI‐lowering association for the minor allele homozygotes of the other SNPs did not attain significance (Table 2).

**Table 2:** Conditional associations with BMI in a mega sample of 27,863 individuals when all four SNPs are included in a genotypic model

Polymorphism	Heterozygote			Minor allele homozygote		
	*β*	*SE*	*p*‐value	*β*	*SE*	*p*‐value
rs2075650	−0.83	0.34	1.35E‐02	−0.33	1.03	7.48E‐01
rs157580	−0.56	0.23	1.53E‐02	−0.19	0.33	5.65E‐01
rs429358	−0.90	0.34	7.61E‐03	−2.27	1.02	2.59E‐02
rs7412	0.57	0.29	4.81E‐02	−1.25	1.30	3.39E‐01

Note

Major allele homozygous genotype was the reference.

### 2.4 Polygenic associations

**Figure 3:**
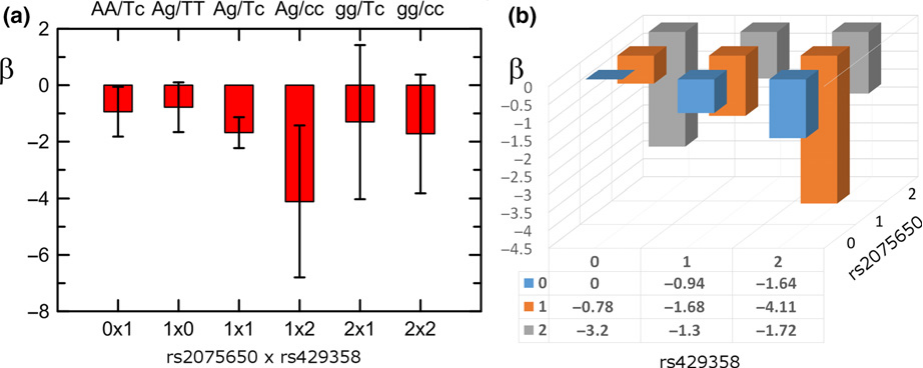
Associations of compound genotypes composed of rs2075650 and rs429358 SNPs with BMI. (a) Effect sizes *β* and 95% confidence intervals for the most frequent compound genotypes. (b) Effect sizes *β* for all compound genotypes. Letters denote rs2075650/rs429358 genotypes. Upper‐ (lower‐) case letter denotes major (minor) allele. Common AA/TT genotype was the reference. Numbers 0, 1, and 2 code the number of minor alleles

Mega‐analysis of compound genotypes composed of rs2075650 and rs429358 showed that the AA/Tc and Ag/TT genotypes were independently associated with BMI at nominal (*β *= −0.94, *p* = 3.63 × 10^−2^) and suggestive (*β *= −0.78, *p* = 8.67 × 10^−2^) levels of significance, respectively (Figure 3 and Supporting Information Table S8). The effect of the Ag/Tc heterozygous genotype was an additive (linear) superposition of the effects of the rs2075650_Tc and rs429358_Ag heterozygotes (*β *= −1.68, *p* = 3.00 × 10^−9^). Subjects carrying the Ag/cc compound genotype had the strongest BMI‐lowering association (*β *= −4.11, *p* = 2.78 × 10^−3^). This result is in qualitative agreement with the result of mega‐analysis of polygenic score composed of these SNPs, which identified the strongest effect for carriers of three minor alleles (*β *= −2.72, *p* = 5.59 × 10^−3^; Supporting Information Table S9, Model 1). However, specificity of the analyses of the polygenic score was poorer compared with the analyses of compound genotypes, as evidenced by smaller effect size in the latter case. Polygenic score composed of rs2075650, rs429358, and rs157580 SNPs supported significant BMI‐lowering associations for carriers of three (*β *= −2.29, *p* = 3.19 × 10^−8^) and four (*β *= −2.33, *p* = 3.08 × 10^−2^) minor alleles (Supporting Information Table S9, Model 2).

### 2.5 Independent associations of rs2075650, rs157580, and *APOE* variants with BMI in younger and older individuals

**Figure 4:**
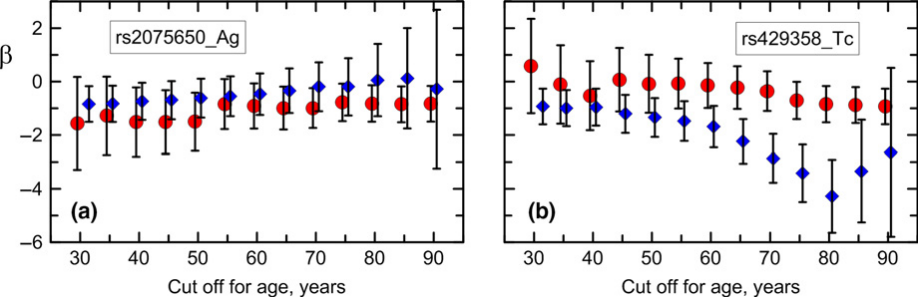
Independent associations of genetic variants with BMI in younger and older individuals. Cutoff shown on the *x*‐axis defines younger (red dots) and older (blue diamonds) individuals. Effect sizes *β* and 95% confidence intervals for (a) rs2075650_Ag and (b) rs429358_Tc heterozygotes in conditional analysis with rs2075650, rs157580, rs429358, and rs7412 in the model. Upper‐ (lower‐) case letter denotes major (minor) allele. Major allele homozygote was the reference. Numerical estimates are given in Supporting Information Table S10

Given uncertainty in defining “younger” and “older” people, we used varying cutoffs ranging from reproductive age (30 years) to the oldest‐old age (90 years) in 5‐year increments. We selected 30 and 90 years as global cutoffs because the analysis in groups of even younger and older ages was not sufficiently powered. In conditional analysis, with rs2075650, rs157580, rs429358, and rs7412 in the model, there were no significant differences between the effect estimates in younger and older carriers of the rs2075650 heterozygote at any given cutoff, as evidenced by well‐overlapped confidence intervals (CIs) (Figure 4a). Nevertheless, there was a trend for a decreased magnitude of the effect size with age from *β *= −0.84, *p* = 1.39 × 10^−2^ for individuals older than 30 years to *β *= −0.28, *p* = 8.55 × 10^−1^ for individuals older than 90 years. The effects were virtually age independent for carriers of the rs157580 heterozygote (Supporting Information Table S10).

In contrast, we observed significant differences between the associations of the rs429358 heterozygote with BMI in younger and older individuals starting at age of about 60 years (i.e., *β *= −0.15, CI = −0.99, 0.69, *p* = 7.32 × 10^−1^ for individuals aged 60 years and younger and *β *= −1.69, CI = −2.46, −0.91, *p* = 1.94 × 10^−5^ for those who were older than 60 years; Figure 4b). There was an apparent trend for an increasing magnitude of the effect size for the older heterozygous carriers of rs429358 until the age of about 80 years, independent of rs2075650, rs157580, and rs7412. For example, for the rs429358 heterozygotes who were 30 years and younger, the effect was in positive direction and non‐significant (*β* = 0.58, CI = −1.18, 2.35, *p* = 5.18 × 10^−1^), whereas for those who were older than 80 years it was in negative direction, large, and genome‐wide significant (*β *= −4.28, CI = −5.65, −2.92, *p* = 7.71 × 10^−10^). For individuals aged 85 years and older, the effect becomes smaller, although standard errors increase due to smaller sample sizes. These trends were similar for carriers of the rs429358 minor allele homozygotes (coding the *APOE* ε4ε4 genotype), although confidence intervals for most groups of older and younger subjects overlapped (Supporting Information Table S10). We did not observe significant differences between the effects in younger and older carriers of either the rs7412 genotypes (Supporting Information Table S10). The results were qualitatively the same in the models with the *APOE* genotypes and alleles.

## 3 DISCUSSION

This study presents the results of the largest analysis so far of the associations of *TOMM40* (rs2075650 and rs157580) and *APOE* (rs429358 and rs7412 coding the ε2/ε3/ε4 polymorphism) variants with BMI in seven cohorts of participants ranging in age from early reproductive age (about 20 years) to centenarians. These analyses leveraged longitudinal information on BMI assessed at different ages and addressed the impact of *TOMM40* and *APOE* variants in the age‐aggregated and age‐stratified samples of older and younger individuals defined using varying cutoffs ranging from 30 years (i.e., people who were younger and older than 30 years at BMI assessment) to 90 years.

Our age‐aggregated analysis shows that minor alleles of rs2075650 and rs429358 are individually associated with smaller BMI (*β *= −1.29, *p* = 3.97 × 10^−9^ and *β *= −1.38, *p* = 2.78 × 10^−10^, respectively) and the rs7412 minor allele is associated with larger BMI (*β* = 0.58, *p* = 3.04 × 10^−2^). Rs157580 did not show significant individual association with BMI. This analysis confirms BMI‐lowering association of the rs2075650 minor allele, first reported in Guo et al. (2013).

Given modest LD between rs2075650 and rs429358 (*r* ^2^ = 49% in our sample), the effect of rs2075650 is often considered as a proxy for that of the *APOE* ε4 allele. For example, studies of longevity indicate that strong effect of rs2075650 is likely due to the effect of the *APOE* isoforms (Murabito, Yuan, & Lunetta, 2012). Studies of AD also indicate that *TOMM40* variants are unlikely to have a major effect on AD (Yu et al., 2007), although the role of *TOMM40‐APOE* haplotypes in AD is also acknowledged (Jazwinski et al., 2010; Lescai et al., 2011; Roses et al., 2010). The role of *TOMM40* and *APOE* variants in body fat remains, however, unclear (Guo et al., 2013). Dissecting this role is important because overall body fatness is considered as a modifiable mid‐life risk factor for development of dementia and AD in late life whereas elevated BMI in late life may have beneficial effect on these conditions (Emmerzaal et al., 2015) while underweight may increase risk of progression to AD in older subjects over time (Joo et al., 2018).

Our conditional analysis with rs2075650 and rs429358 identified independent BMI‐lowering associations of their minor alleles, although the effect sizes (*βs*) for both SNPs became substantially smaller (*β *= −0.63, *p* = 3.99 × 10^−2^ and *β *= −0.94, *p* = 2.17 × 10^−3^, respectively). Polygenic mega‐analysis of compound variants identified additive effects of heterozygotes of these two SNPs (*β *= −1.68, *p* = 3.00 × 10^−9^) and the strongest BMI‐lowering risk for carriers of the rs2075650 heterozygote and rs429358 minor allele homozygote (i.e., ε4ε4), *β *= −4.11, *p* = 2.78 × 10^−3^. The latter effect implies that carriers of these genotypes have 1.05 kg/m^2^ lower BMI compared with carriers of the major allele homozygotes. Mega‐analysis of polygenic score indicated the largest BMI‐lowering risks of three minor alleles of these SNPs, although it was smaller than that for compound genotypes. Accordingly, the analysis of compound variants was more useful in this study than that of polygenic score, constructed as sum of SNP‐unspecified minor alleles, because it identified specific genetic profile of people with strong predisposition to lower BMI.

Previous studies reported that *TOMM40‐APOE* variants other than rs2075650 may be involved in regulation of body fat (Lu et al., 2016). Our conditional analysis, with rs2075650, rs157580, rs429358, and rs7412 in the model, identified independent associations of all heterozygous genotypes (despite the lack of significant individual effect of rs157580) with BMI. The rs2075650, rs157580, and rs429358 heterozygotes were independently associated with lower BMI. Mega‐analysis of polygenic score composed of the rs2075650, rs429358, and rs157580 SNPs identified the largest BMI‐lowering risks of virtually the same size for carriers of three (*β *= −2.29, *p* = 3.19 × 10^−8^) and four (*β *= −2.33, *p* = 3.08 × 10^−2^) minor alleles, although these effects were about half of the strongest effect size for the rs2075650/rs429358 compound variants (see above). Consistent with previous studies (Tejedor, Garcia‐Sobreviela, Ledesma, & Arbones‐Mainar, 2014; Volcik et al., 2006), the rs7412 heterozygote was associated with higher BMI in the current analysis.

*TOMM40* encodes a protein localized in the outer membrane of the mitochondria, which is a part of a complex for mitochondrial protein import (Endo & Yamano, 2010). Mitochondria are a major player in ATP production, maintaining energy balance, and disposal of reactive oxygen species. Excessive energy can disrupt mitochondrial function that affects lipid and glucose metabolism (Bournat & Brown, 2010). Disruption of mitochondrial transport, followed by mitochondrial dysfunction, might be hypothesized as a plausible mechanism for *TOMM40* in regulation of adipocyte function and overall body fatness (Gómez‐Serrano et al., 2017). Studies suggest that APOE, a key protein in lipid metabolism, plays a role in adipocyte function. APOE has been implicated in the development of diet‐induced obesity (Elosua et al., 2003; Feitosa et al., 2006; Kypreos et al., 2009). The *APOE* isoforms can be involved in regulation of body fatness through differences in clearance of dietary fat (Kolovou, Damaskos, Anagnostopoulou, & Cokkinos, 2009; Koopal, van derGraaf, Asselbergs, Westerink, & Visseren, 2015). Mice studies suggest differential role of the *APOE* isoforms in digesting dietary energy (Arbones‐Mainar, Johnson, Altenburg, & Maeda, 2008; Huebbe et al., 2015; Kuhel et al., 2013). The mechanism of influence of APOE on body fat traits might be through modulation on triglyceride content of adipocytes (Kypreos et al., 2009; Li & Liu, 2014).

Our age‐stratified conditional analysis with rs2075650, rs157580, rs429358, and rs7412 in the model revealed well‐powered support for a differential association of the *APOE* ε4‐coding rs429358 variant with BMI in younger and older individuals, independent of rs2075650, rs157580, and rs7412. The effect of the rs429358 heterozygous genotype is most pronounced in individuals aged 60–80 years. No significant effect of this variant was observed in younger people in their early reproductive life (e.g., *β* = 0.58, CI = −1.18, 2.35, *p* = 5.18 × 10^−1^ for people aged ≤30 years, *N* = 3,068) but it was strong and robust in older people (e.g., *β *= −4.28, CI = −5.65, −2.92, *p* = 7.71 × 10^−10^ for people aged >80 years, *N* = 6,052). Few previous studies examining associations of the *APOE* isoforms with body fat traits reported associations of the ε4 allele with decreased obesity in less‐fitted children (Ellis et al., 2011) and non‐significant trend in mid‐aged adults (Volcik et al., 2006). Our previous study identified lower BMI in older ε4 allele carriers compared with the non‐carriers in the FHS original cohort at ages >65 years (Yashin et al., 2013). The differential role of the *APOE* ε4 allele in BMI in younger and older individuals is consistent with differential role of the ε4 allele in total cholesterol with age (Kulminski et al., 2013) and with change of frequency of this allele with birth years (Nygaard et al., 2014). This is also in line with age‐specific associations of the ε4 allele with survival (Jacobsen et al., 2010; Tan et al., 2013), although this effect can be sex‐specific (Joshi et al., 2016; Kulminski et al., 2014). No significant age‐dependent associations of the *TOMM40* SNPs and the *APOE* ε2 allele with BMI, that is difference in younger and older individuals, were identified. Although, there was a non‐significant trend on decreased magnitude of the effect for the rs2075650 older heterozygotes.

Our results on the differential role of the *APOE* ε4 allele in BMI in younger and older individuals contribute to better understanding of genetic architecture underlying differential role of elevated mid‐ and late‐life BMI in risks of AD and dementia (Emmerzaal et al., 2015). They suggest that the ε4 allele may provide late‐life‐specific contribution to increased risk of AD through the mechanism of regulation of body fat, as discussed above, that is consistent with increasing risk of AD with age in general population (Akushevich, Kravchenko, Ukraintseva, Arbeev, & Yashin, 2012) and higher risk for underweight subjects to develop AD in old age (Joo et al., 2018). These results warrant comprehensive longitudinal analysis of dynamic connections between body fat metabolism and progression to AD over the individuals’ life course. Such analysis is necessary to examine genetic predisposition to complex interplay of changes in the body composition with age, aging process, and time trends in obesity and incidence of AD.

The results for the *APOE* ε2/ε3/ε4 polymorphism resembled those for rs429358 and rs7412 SNPs. Examination of rs7412 was, however, more beneficial in this study because of the ability to better handle the associations for the ε2 and ε4 alleles.

Despite rigor of this study, there are potential limitations. First, although this is the largest study of additive effects of the *TOMM40‐APOE* locus SNPs to date, more accurate determination of associations for minor allele homozygous carriers and the associations at extreme ages requires larger samples. Second, we did not investigate the effect of secular trends in BMI, compositional changes in the studied cohorts during follow‐up, and potential role of behavioral factors.

Thus, in addition to confirmation of the BMI‐lowering association of the rs2075650 minor allele, this study provided three major insights. First, it identified that despite modest LD, rs2075650 and rs429358 SNPs were independently associated with lower levels of BMI. Second, the associations of these SNPs were of additive (i.e., complementary) nature. Third, this study revealed that the *APOE* ε4‐coding rs429358 SNP was associated with BMI in older individuals but not in younger individuals, independently of rs2075650, rs157580, and rs7412 SNPs. These results signify independent and additive roles of the *APOE* and *TOMM40* genes in body fat regulation that indicates different (i.e., the *APOE‐* and *TOMM40*‐related) mechanisms of such regulation in this locus. They also signify potential age‐sensitivity of the *APOE*‐related mechanism as the relationship of the *APOE* ε4 allele to BMI differs in younger and older subjects.

## 4 EXPERIMENTAL PROCEDURES

### 4.1 Accession numbers

This manuscript was prepared using a limited access datasets obtained though dbGaP. The dbGaP accession numbers are as follows: phs000007.v22.p8 (FHS), phs000280.v2.p1 (ARIC), phs000209.v12.p3 (MESA), phs000287.v3.p1 (CHS), phs000285.v3.p2 (CARDIA), and phs000428.v1.p1 (HRS). Phenotypic HRS data are available publicly and through restricted access from the University of Michigan https://hrsonline.isr.umich.edu/index.php?p = data.

### 4.2 Study cohorts

Data were obtained from seven longitudinal studies from the Atherosclerosis Risk in Communities (ARIC) study (Investigators, 1989), Cardiovascular Health Study (CHS) (Fried et al., 1991), Coronary Artery Risk Development in Young Adults (CARDIA) study (Friedman et al., 1988), Multi‐Ethnic Study of Atherosclerosis (MESA) (Blind et al., 2002), Health and Retirement Study (HRS) (Juster & Suzman, 1995), Long Life Family Study (LLFS) (Sebastiani et al., 2009), and Framingham Heart Study (FHS) (Splansky et al., 2007) cohorts for individuals of Caucasian ancestry. The FHS included three cohorts comprising parental (FHS_C1), offspring (FHS_C2), and grandchildren (FHS_C3) generations. Basic demographic information for the genotyped participants in the selected studies is provided in Supporting Information Table S1.

### 4.3 Genotypes

Rs2075650 and rs157580 SNPs from *TOMM40* gene were available from Affymetrix (1 M SNPs) chip in the ARIC and MESA, Illumina CVDSNP55v1_A (50 K SNPs) chip in the CARDIA, CHS, and FHS, and Illumina HumanOmni 2.5 Quad chip (2.5 M SNPs) in the HRS and LLFS. Two SNPs coding the *APOE* ε2/ε3/ε4 polymorphism (ε2ε2, ε2ε3, ε2ε4, ε3ε3, ε3ε4, ε4ε4), rs7412 and rs429358, were available from Illumina Hiseq 2000 in ARIC and MESA, and the Taqman array in the LLFS. *APOE* in CARDIA, CHS, FHS_C1, and FHS_C2 were genotyped directly. Rs7412 and rs429358 for participants of the HRS and FHS_C3 were imputed (IMPUTE2) according to the 1000 Genomes Project Phase I integrated variant set release (SHAPEIT2) in the NCBI build 37 (hg19) coordinate with high accuracy (info >0.9).

The rs7412 and rs429358 minor alleles code the *APOE* ε2 and ε4 alleles, respectively (Supporting Information Table S2). This relationship is useful to unambiguously assign the *APOE* ε2ε4 genotype coded by the rs7412 and rs429358 heterozygotes. For comparison, we also defined the ε2 allele as ε2/ε2 or ε2/ε3 genotypes and the ε4 allele as ε3/ε4 or ε4/ε4 genotype excluding the ε2/ε4 genotype from definition of the ε2 or ε4 carrier status. Genotyping information for all polymorphisms is presented in Supporting Information Table S3.

Missing information on SNPs was excluded to have balanced samples with the same number of subjects for each SNP. Polygenic scores were defined as unweighted counts of minor alleles.

### 4.4 Analysis

Measurements of BMI were natural‐log‐transformed to offset potential bias due to skewness of their frequency distributions and multiplied by 100 for better resolution. BMI was measured multiple times during follow‐up of the same individuals in most cohorts (Supporting Information Table S1). We used all available measurements. Information on longitudinal measurements has multiple advantages including potential gain in statistical power in the analyses (Shi, Rice, Gu, & Rao, 2009). The analyses were performed in each cohort separately and the mega sample of all cohorts combined. To correct for repeated‐measurements (all cohorts) and familial (FHS and LLFS) correlations, we used the linear mixed effects model (*lme4* package in R). We evaluated the associations for SNPs given the measurements of BMI for individuals of a given age at each examination with available measurements. Because of longitudinal follow‐up, the same individuals were used in age‐stratified analyses in “old” and/or “young” group depending on his/her age at BMI assessment. The models were adjusted for sex and birth cohorts. Potential inter‐study differences were handled by using mixed effects for studies. The models were not adjusted for principal components to control for population stratification in these Caucasian populations because of their trivial effects in these analyses.

## CONFLICT OF INTEREST

The authors declare that they have no conflict of interest.

## AUTHOR CONTRIBUTIONS

A.M.K. conceived and designed the experiment and wrote the paper; Y.L. performed statistical analyses and contributed to drafting of the paper; I.C. prepared data and performed bioinformatics analysis; J.H., K.G.A., and O.B. prepared data; M.F.F. J.M.Z., K.C., and A.I.Y. contributed to discussion of the intermediate and final results and drafting the manuscript.
