# Supplementary material for: Independent associations of TOMM40 and APOE variants with body mass index
Source: Aging Cell. 2018 Nov 21;18(1):e12869. doi: 10.1111/acel.12869 (PMC6351823; doi:10.1111/acel.12869)
Supplement: Supplementary file 1 [file ACEL-18-e12869-s001.docx]

**Independent associations of *TOMM40* and *APOE* variants with body mass index**

Alexander M. Kulminski^1^, Yury Loika^1^, Irina Culminskaya^1^, Jian Huang^1^, Konstantin G. Arbeev^1^, Olivia Bagley^1^, Mary F. Feitosa^2^, Joseph M. Zmuda^3^, Kaare Christensen^4^, Anatoliy I. Yashin^1^, and the Long Life Family Study research group.

^1^Biodemography of Aging Research Unit, Social Science Research Institute, Duke University, Durham, NC 27708-0408, USA.

^2^Division of Statistical Genomics, Department of Genetics, Washington University School of Medicine, 520 South Euclid Avenue, Campus Box 8506-98-601, St Louis, MO 63110-1093, USA

^3^ Department of Epidemiology, Graduate School of Public Health, University of Pittsburgh, 130 De Soto St, Pittsburgh, PA 15261, USA

^4^The Danish Aging Research Center, University of Southern Denmark, 5000 Odense C, Denmark

**SUPPORTING INFORMATION**

Supporting information includes one Text and ten Tables.

| **1.** | **Supporting Acknowledgment Text**  **Text S1**  Supporting Acknowledgement Text. | **3** |
| --- | --- | --- |
| **2.** | **Supporting Tables** | **5** |
|  | **Table S1**  Basic demographic information for the genotyped participants in the selected studies. | 5 |
|  | **Table S2**  Coding of the *APOE* common polymorphism. | 6 |
|  | **Table S3**  Genotyping information. | 7 |
|  | **Table S4**  Contingency tables for the selected polymorphisms. | 8 |
|  | **Table S5**  Associations of rs2075650 polymorphism with BMI of carriers of APOE alleles in a mega sample of 27,863 individuals from seven longitudinal studies. | 10 |
|  | **Table S6**  Univariate and multivariate associations of selected polymorphisms with BMI in a mega sample of 27,863 individuals from seven longitudinal studies conditional on the rs157580 polymorphism. | 11 |
|  | **Table S7**  Multivariate associations of selected polymorphisms with BMI in a mega sample of 27,863 individuals from seven longitudinal studies. | 12 |
|  | **Table S8**  Associations of compound genotypes composed of rs2075650 and rs429358 SNPs with BMI in a mega sample of 27,863 individuals from seven longitudinal studies. | 13 |
|  | **Table S9**  Associations of polygenic scores accounting for sum of minor alleles of rs2075650, rs429358 and rs157580 SNPs with BMI in a mega sample of 27,863 individuals from seven longitudinal studies. | 14 |
|  | **Table S10**  Multivariate associations of rs2075650, rs157580, rs429358, and rs7412 with BMI in younger and older individuals in the mega sample of 27,863 individuals from seven longitudinal studies. | 15 |
|  |  |  |

**1. Supporting Acknowledgment Text**

**Text S1:** Supporting Acknowledgment Text.

This work was supported by the National Institute on Aging (NIA) (grant numbers U01 AG023712, P01 AG043352, R01 AG047310), NIH, USA. The Long Life Family Study is funded by U01AG023749, U01AG023744, U01AG049508 and U01AG023712 from the NIA. The funding source did not participate in the writing of the manuscript or the decision to submit the manuscript for publication. The content is solely the responsibility of the authors and does not necessarily represent the official views of the National Institutes of Health (NIH). This manuscript was prepared using a limited access datasets obtained though dbGaP. The dbGaP accession numbers are as follows: phs000007.v22.p8 (FHS), phs000280.v2.p1 (ARIC), phs000209.v12.p3 (MESA), phs000287.v3.p1 (CHS), phs000285.v3.p2 (CARDIA), phs000428.v1.p1 (HRS). Phenotypic HRS data are available publicly and through restricted access from the University of Michigan <http://hrsonline.isr.umich.edu/index.php?p=data>.

The Framingham Heart Study (FHS) is conducted and supported by the National Heart, Lung, and Blood Institute (NHLBI) in collaboration with Boston University (Contract No. N01-HC-25195 and HHSN268201500001I). This manuscript was not prepared in collaboration with investigators of the FHS and does not necessarily reflect the opinions or views of the FHS, Boston University, or NHLBI. Funding for SHARe Affymetrix genotyping was provided by NHLBI Contract N02-HL-64278. SHARe Illumina genotyping was provided under an agreement between Illumina and Boston University. Funding for CARe genotyping was provided by NHLBI Contract N01-HC-65226. Funding support for the Framingham Dementia dataset was provided by NIH/NIA grant R01 AG08122.

The Atherosclerosis Risk in Communities Study (ARIC) is carried out as a collaborative study supported by the NHLBI contracts (HHSN268201100005C, HHSN268201100006C, HHSN268201100007C, HHSN268201100008C, HHSN268201100009C, HHSN268201100010C, HHSN268201100011C, and HHSN268201100012C). The authors thank the staff and participants of the ARIC study for their important contributions. Funding for GENEVA was provided by the National Human Genome Research Institute grant U01HG004402 (E. Boerwinkle).

Multi-Ethnic Study of Atherosclerosis (MESA) and the MESA SHARe project are conducted and supported by the NHLBI in collaboration with MESA investigators. Support for MESA is provided by contracts N01-HC-95159, N01-HC-95160, N01-HC-95161, N01-HC-95162, N01-HC-95163, N01-HC-95164, N01-HC-95165, N01-HC-95166, N01-HC-95167, N01-HC-95168, N01-HC-95169 and CTSA UL1-RR-024156. Funding for SHARe genotyping was provided by NHLBI Contract N02-HL-64278. This manuscript was not prepared in collaboration with MESA investigators and does not necessarily reflect the opinions or views of MESA, or the NHLBI.

The Cardiovascular Health Study (CHS) was supported by contracts HHSN268201200036C, HHSN268200800007C, N01-HC-85079, N01-HC-85080, N01-HC-85081, N01-HC-85082, N01-HC-85083, N01-HC-85084, N01-HC-85085, N01-HC-85086, N01-HC-35129, N01 HC-15103, N01 HC-55222, N01-HC-75150, N01-HC-45133, and N01-HC-85239; grant numbers U01 HL080295 and U01 HL130014 from the National Heart, Lung, and Blood Institute (NHLBI), and R01 AG-023629 from the National Institute on Aging, with additional contribution from the National Institute of Neurological Disorders and Stroke. A full list of principal CHS investigators and institutions can be found at https://chs-nhlbi.org/pi. This manuscript was not prepared in collaboration with CHS investigators and does not necessarily reflect the opinions or views of CHS, or the NHLBI. Additional support for infrastructure was provided by HL105756 and additional genotyping among the African-American cohort was supported in part by HL085251. DNA handling and genotyping at Cedars-Sinai Medical Center was supported in part by National Center for Research Resources grant UL1RR033176, now at the National Center for Advancing Translational Technologies CTSI grant UL1TR000124; in addition to the National Institute of Diabetes and Digestive and Kidney Diseases grant DK063491 to the Southern California Diabetes Endocrinology Research Center.

The Coronary Artery Risk Development in Young Adults Study (CARDIA) is conducted and supported by the National Heart, Lung, and Blood Institute (NHLBI) in collaboration with the University of Alabama at Birmingham (N01-HC95095 & N01-HC48047), University of Minnesota (N01-HC48048), Northwestern University (N01-HC48049), and Kaiser Foundation Research Institute (N01-HC48050). This manuscript was not approved by CARDIA. The opinions and conclusions contained in this publication are solely those of the authors, and are not endorsed by CARDIA or the NHLBI and should not be assumed to reflect the opinions or conclusions of either. Genotyping for the CARDIA GENEVA cohort was supported by grant U01 HG004729 from the National Human Genome Research Institute.

The Health and Retirement Study (HRS) genetic data is sponsored by the Genetics Resource with HRS April 21, 2010, version G Page 5 of 7 National Institute on Aging (grant numbers U01AG009740, RC2AG036495, and RC4AG039029) and was conducted by the University of Michigan. This manuscript was not prepared in collaboration with HRS investigators and does not necessarily reflect the opinions or views of HRS.

**2. Supporting Tables**

**Table S1**. Basic demographic information for the genotyped participants in the selected studies.

| Study | N | BMI*, kg/m^2^  mean (SD) | Men (%) | Birth year, mean (SD) | Birth year, range | Age at baseline, mean (SD) | Number of visits |
| --- | --- | --- | --- | --- | --- | --- | --- |
| ARIC | 408 | 27.31 (5.02) | 214 (52.45) | 1933.64 (5.69) | 1922–1944 | 53.89 (5.60) | 4 |
| CARDIA | 1,362 | 23.60 (3.98) | 644 (47.28) | 1957.46 (3.47) | 1950–1965 | 25.62 (3.34) | 6 |
| CHS | 4,008 | 26.29 (4.43) | 1,744 (43.51) | 1914.10 (5.75) | 1890–1925 | 72.88 (5.62) | 3 |
| HRS | 9,358 | 27.63 (5.64) | 3,937 (42.07) | 1938.16 (10.48) | 1905–1975 | 69.65 (10.96) | 12 |
| FHS_1 | 636 | 24.11 (3.45) | 211 (33.18) | 1911.79 (4.18) | 1895–1920 | 35.81 (4.34) | 22 |
| FHS_2 | 3,059 | 25.08 (4.19) | 1,405 (45.93) | 1935.74 (9.62) | 1910–1965 | 34.77 (9.78) | 8 |
| FHS_3 | 3,951 | 26.90 (5.56) | 1,855 (46.95) | 1960.46 (8.94) | 1930–1980 | 40.16 (8.83) | 1 |
| MESA | 703 | 27.78 (5.29) | 325 (46.23) | 1939.46 (10.44) | 1917–1957 | 61.55 (10.43) | 5 |
| LLFS | 4,378 | 27.18 (5.29) | 1,980 (45.23) | 1937.35 (15.63) | 1896–1983 | 69.98 (15.55) | 2 |
| Total | 27,863 | 26.71 (5.26) | 12,315 (44.20) | 1937.78 (17.17) | 1890–1983 | 58.80 (19.37) |  |

N denotes genotyped sample after excluding individuals with missing genotyping and phenotyping information.

ARIC = the Atherosclerosis Risk in Communities study, CARDIA = Coronary Artery Risk Development in Young Adults study, CHS = the Cardiovascular Health Study; HRS = the Health and Retirement Study; FHS_1 = the Framingham Heart Study (FHS) original cohort; FHS_2 = the FHS offspring cohort, FHS_3 = the FHS 3^rd^ generation cohort, MESA = Multi-Ethnic Study of Atherosclerosis, LLFS = Long Life Family Study.

*Body mass index (BMI) is representatively shown at baseline or first available examination.

SD = standard deviation.

**Table S2**. Coding of the *APOE* common polymorphism.

|  | rs7412_CC | rs7412_Ct | rs7412_tt |
| --- | --- | --- | --- |
| rs429358_TT | ε3/ε3 | ε2/ε3 | ε2/ε2 |
| rs429358_Tc | ε3/ε4 | ε2/ε4 | -- |
| rs429358_cc | ε4/ε4 | -- | -- |

*APOE*=apolipoprotein E;

Upper (lower) case letters after SNP ID denote major (minor) allele.

**Table S3**. Genotyping information.

|  | Total | ARIC | CARDIA | CHS | HRS | FHS_1 | FHS_2 | FHS_3 | MESA | LLFS |
| --- | --- | --- | --- | --- | --- | --- | --- | --- | --- | --- |
| Sample | 27,863 | 408 | 1,362 | 4,008 | 9,358 | 636 | 3,059 | 3,951 | 703 | 4,378 |
| Rs2075650 genotypes | | | | | | | | | | |
| AA | 76.22 | 70.83 | 73.57 | 76.95 | 74.58 | 81.76 | 75.74 | 76.28 | 74.96 | 80.03 |
| Ag | 22.22 | 26.96 | 24.74 | 21.78 | 23.50 | 17.30 | 22.79 | 22.10 | 23.33 | 18.94 |
| gg | 1.56 | 2.21 | 1.69 | 1.27 | 1.92 | 0.94 | 1.47 | 1.62 | 1.71 | 1.03 |
| MAF | 12.67 | 15.59 | 14.06 | 12.16 | 13.67 | 9.59 | 12.43 | 12.67 | 13.38 | 13.66 |
| *p_HWE_* | 0.345 | 0.852 | 0.433 | 0.176 | 0.804 | 0.360 | 0.875 | 0.943 | 0.869 | 0.402 |
| Rs157580 genotypes | | | | | | | | | | |
| AA | 36.18 | 34.80 | 36.42 | 35.43 | 37.51 | 38.05 | 35.01 | 33.71 | 40.97 | 36.09 |
| Ag | 48.60 | 50.00 | 48.53 | 48.90 | 47.74 | 47.33 | 48.71 | 50.19 | 44.95 | 49.31 |
| gg | 15.22 | 15.20 | 15.05 | 15.67 | 14.75 | 14.62 | 16.28 | 16.10 | 14.08 | 14.60 |
| MAF | 39.52 | 40.20 | 39.31 | 40.12 | 38.62 | 38.29 | 40.04 | 41.20 | 36.55 | 37.70 |
| *p_HWE_* | 0.511 | 0.417 | 0.570 | 0.270 | 0.481 | 0.327 | 0.154 | 0.024 | 0.564 | 0.726 |
| Rs7412 genotypes | | | | | | | | | | |
| CC | 84.39 | 86.03 | 85.61 | 83.91 | 84.36 | 86.79 | 85.32 | 85.17 | 84.07 | 82.71 |
| Ct | 14.99 | 12.25 | 13.73 | 15.39 | 15.10 | 12.89 | 14.28 | 14.20 | 14.79 | 16.58 |
| tt | 0.62 | 1.72 | 0.66 | 0.70 | 0.54 | 0.32 | 0.42 | 0.63 | 1.14 | 0.71 |
| MAF | 8.12 | 7.85 | 7.53 | 8.40 | 8.09 | 6.77 | 7.15 | 7.73 | 8.41 | 7.40 |
| *p_HWE_* | 0.547 | 0.002 | 0.436 | 0.917 | 0.686 | 0.129 | 0.794 | 0.738 | 0.130 | 1.000 |
| Rs429358 genotypes | | | | | | | | | | |
| TT | 76.19 | 74.27 | 77.09 | 75.97 | 74.05 | 80.50 | 76.95 | 75.70 | 73.97 | 80.47 |
| Tc | 22.23 | 23.28 | 21.73 | 22.83 | 23.94 | 18.87 | 21.41 | 22.70 | 23.90 | 18.46 |
| cc | 1.58 | 2.45 | 1.18 | 1.20 | 2.01 | 0.63 | 1.64 | 1.60 | 2.13 | 1.07 |
| MAF | 12.70 | 14.09 | 12.05 | 12.62 | 13.98 | 10.07 | 12.13 | 12.95 | 13.96 | 13.87 |
| *p_HWE_* | 0.194 | 0.417 | 0.441 | 0.020 | 0.580 | 0.184 | 0.872 | 0.724 | 0.873 | 0.336 |
| *APOE* genotypes | | | | | | | | | | |
| ε2/ε2 | 0.62 | 1.72 | 0.66 | 0.70 | 0.55 | 0.31 | 0.39 | 0.63 | 1.14 | 0.71 |
| ε2/ε3 | 12.86 | 10.05 | 12.26 | 13.02 | 12.93 | 11.00 | 12.36 | 11.79 | 12.52 | 14.69 |
| ε2/ε4 | 2.13 | 2.21 | 1.47 | 2.37 | 2.17 | 1.89 | 1.93 | 2.40 | 2.28 | 1.90 |
| ε3/ε3 | 62.70 | 62.50 | 64.17 | 62.25 | 60.58 | 69.18 | 64.20 | 63.28 | 60.31 | 65.07 |
| ε3/ε4 | 20.11 | 21.08 | 20.26 | 20.46 | 21.77 | 16.99 | 19.48 | 20.30 | 21.62 | 16.56 |
| ε4/ε4 | 1.58 | 2.44 | 1.18 | 1.20 | 2.00 | 0.63 | 1.64 | 1.60 | 2.13 | 1.07 |
| ε2 | 8.12 | 7.85 | 7.53 | 8.40 | 8.10 | 6.76 | 7.54 | 7.73 | 8.54 | 9.01 |
| ε3 | 79.19 | 78.07 | 80.43 | 78.99 | 77.93 | 83.18 | 80.12 | 79.33 | 77.38 | 80.70 |
| ε4 | 12.70 | 14.09 | 12.05 | 12.62 | 13.97 | 10.07 | 12.35 | 12.95 | 14.08 | 10.30 |

N=sample size after excluding individuals with missing genotyping and phenotypic information.

*APOE*=apolipoprotein E; MAF=minor allele frequency; *p_HWE_*=*p*-value for Hardy-Weinberg Equilibrium.

Upper (lower) case letters denote major (minor) allele.

ARIC = the Atherosclerosis Risk in Communities study, CARDIA = Coronary Artery Risk Development in Young Adults study, CHS = the Cardiovascular Health Study; HRS = the Health and Retirement Study; FHS_1 = the Framingham Heart Study (FHS) original cohort; FHS_2 = the FHS offspring cohort, FHS_3 = the FHS 3^rd^ generation cohort, MESA = Multi-Ethnic Study of Atherosclerosis, LLFS = Long Life Family Study.

Values for genotypes are shown as percentage (%) of participants

**Table S4**. Contingency tables for the selected polymorphisms.

A)

|  | rs429358_TT | rs429358_Tc | rs429358_cc |
| --- | --- | --- | --- |
| rs2075650_AA | 19,653 (70.53) | 1,545 (5.55) | 38 (0.14) |
| rs2075650_Ag | 1,540 (5.53) | 4,498 (16.14) | 154 (0.55) |
| rs2075650_gg | 35 (0.13) | 151 (0.54) | 249 (0.89) |

B)

|  | rs429358_TT | rs429358_Tc | rs429358_cc |
| --- | --- | --- | --- |
| rs157580_AA | 6,393 (22.94) | 3,266 (11.72) | 418 (1.50) |
| rs157580_Ag | 10,635 (38.17) | 2,883 (10.35) | 23 (0.08) |
| rs157580_gg | 4,200 (15.07) | 40 (0.14) | 0 (0.00) |

C)

|  | rs7412_CC | rs7412_Ct | rs7412_tt |
| --- | --- | --- | --- |
| rs2075650_AA | 17,466 (62.69) | 3,598 (12.91) | 172 (0.62) |
| rs2075650_Ag | 5,616 (20.16) | 575 (2.06) | 1 (0.00) |
| rs2075650_gg | 431 (1.55) | 4 (0.01) | 0 (0.00) |

D)

|  | rs7412_CC | rs7412_Ct | rs7412_tt |
| --- | --- | --- | --- |
| rs157580_AA | 8,912 (31.99) | 1,139 (4.09) | 32 (0.11) |
| rs157580_Ag | 11,264 (40.43) | 2,186 (7.85) | 91 (0.33) |
| rs157580_gg | 3,337 (11.98) | 852 (3.06) | 51 (0.18) |

E)

|  | rs157580_AA | rs157580_Ag | rs157580_gg |
| --- | --- | --- | --- |
| rs2075650_AA | 6,301 (22.61) | 10,698 (38.40) | 4,237 (15.21) |
| rs2075650_Ag | 3,346 (12.01) | 2,843 (10.20) | 3 (0.01) |
| rs2075650_gg | 435 (1.56) | 0 (0.00) | 0 (0.00) |

F)

|  | rs7412_CC | rs7412_Ct | rs7412_tt |
| --- | --- | --- | --- |
| rs429358_TT | 17,470 (62.70) | 3,585 (12.87) | 173 (0.62) |
| rs429358_Tc | 5,602 (20.11) | 592 (2.12) | 0 (0.00) |
| rs429358_cc | 441 (1.58) | 0 (0.00) | 0 (0.00) |

G)

| *APOE* genotypes: | ε2ε2 | ε2ε3 | ε2ε4 | ε3ε3 | ε3ε4 | ε4ε4 |
| --- | --- | --- | --- | --- | --- | --- |
| rs2075650_AA | 172 (0.62) | 3,437 (12.34) | 161 (0.58) | 16,044 (57.58) | 1,384 (4.97) | 38 (0.14) |
| rs2075650_Ag | 1 (0.00) | 146 (0.52) | 429 (1.54) | 1,393 (5.00) | 4,069 (14.60) | 154 (0.55) |
| rs2075650_gg | 0 (0.00) | 2 (0.01) | 2 (0.01) | 33 (0.12) | 149 (0.53) | 249 (0.89) |

H)

| *APOE* genotypes: | ε2ε2 | ε2ε3 | ε2ε4 | ε3ε3 | ε3ε4 | ε4ε4 |
| --- | --- | --- | --- | --- | --- | --- |
| rs157580_AA | 31 (0.11) | 905 (3.25) | 234 (0.84) | 5,457 (19.59) | 3,037 (10.90) | 418 (1.50) |
| rs157580_Ag | 91 (0.33) | 1,837 (6.59) | 349 (1.25) | 8,707 (31.25) | 2,534 (9.09) | 23 (0.08) |
| rs157580_gg | 51 (0.18) | 843 (3.03) | 9 (0.03) | 3,306 (11.87) | 31 (0.11) | 0 (0.00) |

Tables show the number and percentage in parentheses of subjects with a given genotype.

Upper (lower) case letters after SNP ID denote major (minor) allele.

*APOE*=apolipoprotein E

**Table S5**. Associations of rs2075650 polymorphism with BMI of carriers of APOE alleles in a mega sample of 27,863 individuals from seven longitudinal studies.

| APOE allele carriers | β | SE | *p*-value |
| --- | --- | --- | --- |
| ε2ε2 | -1.72 | 16.4 | 9.17E-01 |
| ε2ε3 | 0.99 | 1.42 | 4.88E-01 |
| ε2ε4 | -0.36 | 1.60 | 8.20E-01 |
| ε3ε3 | -1.01 | 0.46 | 2.65E-02 |
| ε3ε4 | -0.52 | 0.48 | 2.85E-01 |
| ε4ε4 | 0.68 | 1.35 | 6.16E-01 |

Additive genetic model with minor allele of rs2075650 polymorphism as an effect allele.

**Table S6**. Univariate and multivariate associations of selected polymorphisms with BMI in a mega sample of 27,863 individuals from seven longitudinal studies conditional on the rs157580 polymorphism.

| Polymorphism | Model 1 | | | | Model 2 | | | Model 3 | | | Model 4 | | | Model 5 | | | Model 6 | | |
| --- | --- | --- | --- | --- | --- | --- | --- | --- | --- | --- | --- | --- | --- | --- | --- | --- | --- | --- | --- |
|  | β | SE | *p*-value | β | | SE | *p*-value | β | SE | *p*-value | β | SE | *p*-value | β | SE | *p*-value | β | SE | *p*-value |
| rs2075650* | -1.29 | 0.22 | 3.97E-09 | -1.34 | | 0.23 | 4.91E-09 |  |  |  |  |  |  |  |  |  |  |  |  |
| rs157580* | 0.15 | 0.15 | 3.03E-01 | -0.13 | | 0.16 | 4.22E-01 | -0.13 | 0.16 | 4.04E-01 | 0.12 | 0.15 | 4.12E-01 | -0.15 | 0.16 | 3.48E-01 | -0.12 | 0.16 | 4.44E-01 |
| rs429358* | -1.38 | 0.22 | 2.78E-10 |  | |  |  | -1.43 | 0.23 | 3.34E-10 |  |  |  |  |  |  |  |  |  |
| rs7412* | 0.58 | 0.27 | 3.04E-02 |  | |  |  |  |  |  | 0.56 | 0.27 | 3.82E-02 |  |  |  |  |  |  |
| ε2ε2^†^ | -1.23 | 1.30 | 3.45E-01 |  | |  |  |  |  |  |  |  |  | -1.19 | 1.30 | 3.60E-01 |  |  |  |
| ε2ε3^†^ | 0.55 | 0.31 | 8.26E-02 |  | |  |  |  |  |  |  |  |  | 0.56 | 0.31 | 7.43E-02 |  |  |  |
| ε2ε4^†^ | -0.66 | 0.71 | 3.54E-01 |  | |  |  |  |  |  |  |  |  | -0.70 | 0.71 | 3.28E-01 |  |  |  |
| ε3ε4^†^ | -1.41 | 0.26 | 7.68E-08 |  | |  |  |  |  |  |  |  |  | -1.47 | 0.27 | 5.26E-08 |  |  |  |
| ε4ε4^†^ | -2.41 | 0.82 | 3.32E-03 |  | |  |  |  |  |  |  |  |  | -2.53 | 0.83 | 2.31E-03 |  |  |  |
| ε2^‡^ | 0.46 | 0.31 | 1.36E-01 |  | |  |  |  |  |  |  |  |  |  |  |  | 0.47 | 0.31 | 1.25E-01 |
| ε4^‡^ | -1.48 | 0.26 | 6.50E-09 |  | |  |  |  |  |  |  |  |  |  |  |  | -1.54 | 0.26 | 6.67E-09 |

Model 1: Associations of rs2075650, rs157580, rs429358, rs7412, *APOE* genotypes, and *APOE* alleles separately. The *APOE* ε2 allele was defined as the ε2ε2 or ε2ε3 genotypes. The *APOE* ε4 allele was defined as the ε3ε4 or ε4ε4 genotypes. The ε2/ε4 genotype was excluded from definition of the ε2 or ε4 carrier status.

Model 2: Bivariate model of additive effects of rs157580 and rs2075650 SNPs.

Model 3: Bivariate model of additive effects of rs157580 and rs429358 SNPs.

Model 4: Bivariate model of additive effects of rs157580 and rs7412 SNPs.

Model 5: Multivariate model of additive effects of rs157580 and *APOE* genotypes.

Model 6: Multivariate model of additive effects of rs157580 and *APOE* alleles.

* Additive genetic model with minor allele as an effect allele.

^†^ Genotypic model for *APOE* with the ε3ε3 genotype as a reference.

^‡^ Allelic model for *APOE* with the ε3ε3 genotype as a reference.

**Table S7**. Multivariate associations of selected polymorphisms with BMI in a mega sample of 27,863 individuals from seven longitudinal studies.

| Polymorphism | Model 1 | | | Model 2 | | |
| --- | --- | --- | --- | --- | --- | --- |
|  | β | SE | *p*-value | β | SE | *p*-value |
| rs2075650_1 | -0.83 | 0.34 | 1.34E-02 | -0.84 | 0.35 | 1.46E-02 |
| rs2075650_2 | -0.32 | 1.03 | 7.55E-01 | -1.11 | 0.89 | 2.08E-01 |
| rs157580_1 | -0.56 | 0.23 | 1.50E-02 | -0.52 | 0.23 | 2.54E-02 |
| rs157580_2 | 0.19 | 0.33 | 5.64E-01 | -0.18 | 0.33 | 5.88E-01 |
| ε2ε2 | -1.25 | 1.30 | 3.36E-01 |  |  |  |
| ε2ε3 | 0.53 | 0.32 | 9.25E-02 |  |  |  |
| ε2ε4 | -0.11 | 0.75 | 8.86E-01 |  |  |  |
| ε3ε4 | -0.93 | 0.35 | 7.42E-03 |  |  |  |
| ε4ε4 | -2.29 | 1.02 | 2.51E-02 |  |  |  |
| ε2 |  |  |  | 0.44 | 0.31 | 1.53E-01 |
| ε4 |  |  |  | -0.96 | 0.35 | 5.55E-03 |

Genotypic models with major allele homozygous genotype as a reference for SNPs and the ε3ε3 genotype as a reference for the APOE polymorphism.

The *APOE* ε2 allele was defined as the ε2ε2 or ε2ε3 genotypes.

The *APOE* ε4 allele was defined as the ε3ε4 or ε4ε4 genotypes.

The ε2/ε4 genotype was excluded from definition of the ε2 or ε4 carrier status.

Model 1: Multivariate model of additive effects of rs2075650, rs157580 SNPs and APOE genotypes.

Model 2: Multivariate model of additive effects of rs2075650, rs157580 SNPs and APOE alleles.

**Table S8**. Associations of compound genotypes composed of rs2075650 and rs429358 SNPs with BMI in a mega sample of 27,863 individuals from seven longitudinal studies.

| SNP genotype | | Sample | Model | | |
| --- | --- | --- | --- | --- | --- |
| rs2075650 | rs429358 | N | β | SE | *p*-value |
| 0 | 0 | 19,653 | Reference |  |  |
| 0 | 1 | 1,545 | -0.94 | 0.45 | 3.63E-02 |
| 0 | 2 | 38 | -1.64 | 2.77 | 5.53E-01 |
| 1 | 0 | 1,540 | -0.78 | 0.45 | 8.67E-02 |
| 1 | 1 | 4,498 | -1.68 | 0.28 | 3.00E-09 |
| 1 | 2 | 154 | -4.11 | 1.37 | 2.78E-03 |
| 2 | 0 | 35 | -3.20 | 2.94 | 2.33E-01 |
| 2 | 1 | 151 | -1.30 | 1.39 | 3.50E-01 |
| 2 | 2 | 249 | -1.72 | 1.07 | 1.10E-01 |

Genotypic models with major allele homozygous genotype as a reference.

0/1/2 codes major-homozygous/heterozygous/minor-homozygous genotypes.

**Table S9**. Associations of polygenic scores accounting for sum of minor alleles of rs2075650, rs429358 and rs157580 SNPs with BMI in a mega sample of 27,863 individuals from seven longitudinal studies.

| Number of minor alleles | Model 1 | | | Model 2 | | |
| --- | --- | --- | --- | --- | --- | --- |
|  | β | SE | *p*-value | β | SE | *p*-value |
| 0 | Reference |  |  | Reference |  |  |
| 1 | -0.86 | 0.33 | 9.39E-03 | -0.54 | 0.28 | 5.37E-02 |
| 2 | -1.69 | 0.28 | 1.80E-09 | -0.88 | 0.30 | 3.45E-03 |
| 3 | -2.72 | 0.98 | 5.59E-03 | -2.29 | 0.41 | 3.19E-08 |
| 4 | -1.73 | 1.08 | 1.10E-01 | -2.33 | 1.08 | 3.08E-02 |

Genotypic models with major allele homozygous genotype as a reference.

Model 1: Associations of polygenic score constructed by summation of numbers of minor alleles of two SNPs, rs2075650 and rs429358.

Model 2: Associations of polygenic score constructed by summation of numbers of minor alleles of three SNPs, rs2075650, rs429358 and rs157580.

**Table S10**. Multivariate associations of rs2075650, rs157580, rs429358, and rs7412 with BMI in younger and older individuals in the mega sample of 27,863 individuals from seven longitudinal studies.

|  |  | Age30 | | | Age35 | | | Age40 | | | Age45 | | | Age50 | | |
| --- | --- | --- | --- | --- | --- | --- | --- | --- | --- | --- | --- | --- | --- | --- | --- | --- |
|  |  | Nind | | Nobs | Nind | | Nobs | Nind | | Nobs | Nind | | Nobs | Nind | | Nobs |
| Sample size | young | 3,068 | | 5,503 | 4,590 | | 10,018 | 6,208 | | 15,064 | 7,885 | | 20,382 | 10,018 | | 26,866 |
|  | old | 27,266 | | 139,484 | 26,512 | | 134,969 | 25,155 | | 129,923 | 23,537 | | 124,605 | 22,558 | | 118,121 |
|  | TOTAL | 30,334 | | 144,987 | 31,102 | | 144,987 | 31,363 | | 144,987 | 31,422 | | 144,987 | 32,576 | | 144,987 |
| Polymorphism | Sample | β | SE | *p*-value | β | SE | *p*-value | β | SE | *p*-value | β | SE | *p*-value | β | SE | *p*-value |
| rs2075650_Ag | young | -1.56 | 0.89 | 7.79E-02 | -1.28 | 0.75 | 8.67E-02 | -1.51 | 0.66 | 2.21E-02 | -1.51 | 0.61 | 1.27E-02 | -1.50 | 0.55 | 6.61E-03 |
| rs2075650_Ag | old | -0.84 | 0.34 | 1.39E-02 | -0.83 | 0.35 | 1.61E-02 | -0.74 | 0.35 | 3.69E-02 | -0.70 | 0.36 | 5.55E-02 | -0.62 | 0.37 | 9.52E-02 |
| rs2075650_gg | young | 0.21 | 2.74 | 9.38E-01 | 1.75 | 2.36 | 4.58E-01 | 0.59 | 2.09 | 7.78E-01 | -0.82 | 1.90 | 6.66E-01 | -1.12 | 1.73 | 5.16E-01 |
| rs2075650_gg | old | -0.41 | 1.05 | 6.94E-01 | -0.69 | 1.06 | 5.16E-01 | -0.83 | 1.09 | 4.44E-01 | -0.39 | 1.12 | 7.26E-01 | -0.08 | 1.15 | 9.44E-01 |
| rs157580_Ag | young | -0.69 | 0.65 | 2.89E-01 | -0.30 | 0.53 | 5.72E-01 | -0.62 | 0.47 | 1.84E-01 | -0.71 | 0.43 | 9.81E-02 | -0.67 | 0.39 | 8.55E-02 |
| rs157580_Ag | old | -0.59 | 0.23 | 1.19E-02 | -0.64 | 0.24 | 6.84E-03 | -0.61 | 0.24 | 1.25E-02 | -0.63 | 0.25 | 1.21E-02 | -0.61 | 0.25 | 1.61E-02 |
| rs157580_gg | young | 0.15 | 0.92 | 8.67E-01 | -0.67 | 0.76 | 3.79E-01 | -0.31 | 0.67 | 6.37E-01 | 0.04 | 0.61 | 9.54E-01 | 0.69 | 0.55 | 2.11E-01 |
| rs157580_gg | old | -0.31 | 0.34 | 3.63E-01 | -0.40 | 0.34 | 2.37E-01 | -0.42 | 0.35 | 2.23E-01 | -0.36 | 0.36 | 3.14E-01 | -0.36 | 0.36 | 3.20E-01 |
| rs429358_Tc | young | 0.58 | 0.90 | 5.18E-01 | -0.11 | 0.75 | 8.87E-01 | -0.53 | 0.66 | 4.24E-01 | 0.07 | 0.61 | 9.08E-01 | -0.08 | 0.55 | 8.81E-01 |
| rs429358_Tc | old | -0.93 | 0.34 | 6.11E-03 | -0.99 | 0.34 | 4.00E-03 | -0.96 | 0.35 | 6.66E-03 | -1.21 | 0.36 | 8.71E-04 | -1.34 | 0.37 | 2.77E-04 |
| rs429358_cc | young | 1.10 | 2.89 | 7.05E-01 | -0.08 | 2.30 | 9.72E-01 | 0.64 | 2.11 | 7.61E-01 | 1.08 | 1.89 | 5.69E-01 | 1.45 | 1.69 | 3.91E-01 |
| rs429358_cc | old | -2.30 | 1.04 | 2.68E-02 | -2.26 | 1.05 | 3.15E-02 | -2.73 | 1.07 | 1.07E-02 | -3.24 | 1.09 | 3.04E-03 | -3.84 | 1.11 | 5.75E-04 |
| rs7412_Ct | young | 1.02 | 0.83 | 2.18E-01 | 0.61 | 0.68 | 3.72E-01 | 0.53 | 0.59 | 3.71E-01 | 0.27 | 0.54 | 6.22E-01 | 0.74 | 0.49 | 1.36E-01 |
| rs7412_Ct | old | 0.59 | 0.29 | 4.37E-02 | 0.56 | 0.30 | 6.25E-02 | 0.55 | 0.31 | 7.10E-02 | 0.62 | 0.31 | 4.60E-02 | 0.55 | 0.32 | 8.62E-02 |
| rs7412_tt | young | -0.61 | 4.25 | 8.86E-01 | -2.48 | 3.74 | 5.07E-01 | -2.40 | 3.64 | 5.10E-01 | -3.83 | 2.77 | 1.66E-01 | -3.24 | 2.17 | 1.37E-01 |
| rs7412_tt | old | -1.20 | 1.31 | 3.59E-01 | -1.22 | 1.33 | 3.60E-01 | -1.32 | 1.34 | 3.27E-01 | -0.44 | 1.41 | 7.55E-01 | -0.71 | 1.43 | 6.18E-01 |

**Table S10**. (continued)

|  |  | Age55 | | | Age60 | | | Age65 | | | Age70 | | | Age75 | | |
| --- | --- | --- | --- | --- | --- | --- | --- | --- | --- | --- | --- | --- | --- | --- | --- | --- |
|  |  | Nind | | Nobs | Nind | | Nobs | Nind | | Nobs | Nind | | Nobs | Nind | | Nobs |
| Sample size | young | 14,338 | | 39,922 | 17,444 | | 59,643 | 19,209 | | 79,143 | 22,068 | | 98,180 | 24,764 | | 116,622 |
|  | old | 21,430 | | 105,065 | 19,493 | | 85,344 | 16,781 | | 65,844 | 13,928 | | 46,807 | 9,843 | | 28,365 |
|  | TOTAL | 35,768 | | 144,987 | 36,937 | | 144,987 | 35,990 | | 144,987 | 35,996 | | 144,987 | 34,607 | | 144,987 |
| Polymorphism | Sample | β | SE | *p*-value | β | SE | *p*-value | β | SE | *p*-value | β | SE | *p*-value | β | SE | *p*-value |
| rs2075650_Ag | young | -0.84 | 0.48 | 7.84E-02 | -0.91 | 0.43 | 3.42E-02 | -0.99 | 0.41 | 1.53E-02 | -0.99 | 0.38 | 9.26E-03 | -0.78 | 0.36 | 2.92E-02 |
| rs2075650_Ag | old | -0.55 | 0.38 | 1.48E-01 | -0.47 | 0.40 | 2.34E-01 | -0.34 | 0.43 | 4.22E-01 | -0.20 | 0.47 | 6.73E-01 | -0.20 | 0.55 | 7.15E-01 |
| rs2075650_gg | young | -1.61 | 1.47 | 2.76E-01 | -0.12 | 1.29 | 9.23E-01 | -0.23 | 1.23 | 8.49E-01 | 0.09 | 1.14 | 9.39E-01 | -0.19 | 1.08 | 8.61E-01 |
| rs2075650_gg | old | -0.35 | 1.17 | 7.66E-01 | -0.43 | 1.23 | 7.29E-01 | -0.14 | 1.32 | 9.16E-01 | -0.04 | 1.47 | 9.76E-01 | 0.25 | 1.77 | 8.87E-01 |
| rs157580_Ag | young | -0.63 | 0.33 | 5.83E-02 | -0.64 | 0.30 | 3.20E-02 | -0.53 | 0.28 | 6.31E-02 | -0.63 | 0.26 | 1.65E-02 | -0.63 | 0.25 | 1.16E-02 |
| rs157580_Ag | old | -0.58 | 0.26 | 2.57E-02 | -0.47 | 0.27 | 8.01E-02 | -0.48 | 0.29 | 9.79E-02 | -0.42 | 0.32 | 1.87E-01 | -0.47 | 0.37 | 2.04E-01 |
| rs157580_gg | young | 0.01 | 0.47 | 9.87E-01 | -0.17 | 0.43 | 6.87E-01 | 0.00 | 0.41 | 9.98E-01 | -0.24 | 0.38 | 5.30E-01 | -0.24 | 0.35 | 5.03E-01 |
| rs157580_gg | old | -0.32 | 0.37 | 3.92E-01 | -0.20 | 0.39 | 6.16E-01 | -0.20 | 0.41 | 6.34E-01 | 0.06 | 0.45 | 8.94E-01 | -0.19 | 0.52 | 7.23E-01 |
| rs429358_Tc | young | -0.08 | 0.48 | 8.73E-01 | -0.15 | 0.43 | 7.32E-01 | -0.22 | 0.41 | 5.81E-01 | -0.36 | 0.38 | 3.46E-01 | -0.71 | 0.36 | 4.76E-02 |
| rs429358_Tc | old | -1.48 | 0.38 | 9.45E-05 | -1.69 | 0.39 | 1.94E-05 | -2.23 | 0.42 | 1.36E-07 | -2.86 | 0.47 | 7.30E-10 | -3.43 | 0.55 | 3.74E-10 |
| rs429358_cc | young | 1.38 | 1.44 | 3.38E-01 | -1.11 | 1.27 | 3.80E-01 | -1.77 | 1.21 | 1.44E-01 | -2.20 | 1.12 | 4.92E-02 | -2.03 | 1.07 | 5.74E-02 |
| rs429358_cc | old | -4.07 | 1.14 | 3.59E-04 | -3.89 | 1.21 | 1.31E-03 | -5.11 | 1.31 | 9.92E-05 | -5.20 | 1.53 | 6.97E-04 | -4.80 | 1.85 | 9.61E-03 |
| rs7412_Ct | young | 0.72 | 0.42 | 8.58E-02 | 0.72 | 0.38 | 5.45E-02 | 0.62 | 0.36 | 8.48E-02 | 0.57 | 0.33 | 8.84E-02 | 0.51 | 0.31 | 1.03E-01 |
| rs7412_Ct | old | 0.42 | 0.33 | 1.98E-01 | 0.28 | 0.34 | 4.12E-01 | 0.56 | 0.36 | 1.21E-01 | 0.70 | 0.39 | 7.47E-02 | 0.83 | 0.46 | 7.08E-02 |
| rs7412_tt | young | -1.88 | 1.92 | 3.27E-01 | -1.54 | 1.76 | 3.81E-01 | -1.72 | 1.69 | 3.11E-01 | -0.80 | 1.55 | 6.05E-01 | -1.37 | 1.44 | 3.41E-01 |
| rs7412_tt | old | -0.75 | 1.47 | 6.12E-01 | -0.60 | 1.56 | 7.00E-01 | -0.72 | 1.62 | 6.57E-01 | -0.96 | 1.73 | 5.77E-01 | -0.90 | 1.95 | 6.44E-01 |

**Table S10**. (continued)

|  |  | Age80 | | | Age85 | | | Age90 | | |
| --- | --- | --- | --- | --- | --- | --- | --- | --- | --- | --- |
|  |  | Nind | | Nobs | Nind | | Nobs | Nind | | Nobs |
| Sample size | young | 26,044 | | 130,502 | 26,670 | | 138,771 | 27,126 | | 142,797 |
|  | old | 6,052 | | 14,485 | 3,263 | | 6,216 | 1,499 | | 2,190 |
|  | TOTAL | 32,096 | | 144,987 | 29,933 | | 144,987 | 28,625 | | 144,987 |
| Polymorphism | Sample | β | SE | *p*-value | β | SE | *p*-value | β | SE | *p*-value |
| rs2075650_Ag | young | -0.82 | 0.35 | 1.86E-02 | -0.85 | 0.34 | 1.32E-02 | -0.83 | 0.34 | 1.46E-02 |
| rs2075650_Ag | old | 0.06 | 0.69 | 9.35E-01 | 0.12 | 0.96 | 8.97E-01 | -0.28 | 1.51 | 8.55E-01 |
| rs2075650_gg | young | -0.27 | 1.06 | 7.98E-01 | -0.30 | 1.05 | 7.74E-01 | -0.35 | 1.04 | 7.37E-01 |
| rs2075650_gg | old | -2.01 | 2.35 | 3.92E-01 | -3.85 | 3.48 | 2.69E-01 | -6.09 | 5.67 | 2.83E-01 |
| rs157580_Ag | young | -0.53 | 0.24 | 2.73E-02 | -0.52 | 0.24 | 2.84E-02 | -0.55 | 0.23 | 1.83E-02 |
| rs157580_Ag | old | -0.75 | 0.46 | 1.01E-01 | -0.16 | 0.61 | 7.97E-01 | 0.51 | 0.92 | 5.79E-01 |
| rs157580_gg | young | -0.21 | 0.34 | 5.44E-01 | -0.17 | 0.34 | 6.10E-01 | -0.21 | 0.34 | 5.39E-01 |
| rs157580_gg | old | -0.17 | 0.65 | 7.92E-01 | -0.36 | 0.84 | 6.68E-01 | 0.47 | 1.26 | 7.10E-01 |
| rs429358_Tc | young | -0.85 | 0.35 | 1.47E-02 | -0.88 | 0.34 | 1.04E-02 | -0.93 | 0.34 | 5.98E-03 |
| rs429358_Tc | old | -4.28 | 0.70 | 7.71E-10 | -3.34 | 0.98 | 6.28E-04 | -2.64 | 1.61 | 1.01E-01 |
| rs429358_cc | young | -2.19 | 1.04 | 3.54E-02 | -2.25 | 1.04 | 3.00E-02 | -2.33 | 1.03 | 2.36E-02 |
| rs429358_cc | old | -5.52 | 2.44 | 2.35E-02 | -3.47 | 3.54 | 3.27E-01 | -1.39 | 6.23 | 8.23E-01 |
| rs7412_Ct | young | 0.44 | 0.30 | 1.43E-01 | 0.54 | 0.30 | 7.27E-02 | 0.61 | 0.30 | 3.99E-02 |
| rs7412_Ct | old | 1.03 | 0.57 | 7.06E-02 | 1.40 | 0.75 | 5.96E-02 | 1.37 | 1.08 | 2.04E-01 |
| rs7412_tt | young | -1.26 | 1.38 | 3.60E-01 | -1.11 | 1.36 | 4.13E-01 | -1.19 | 1.33 | 3.71E-01 |
| rs7412_tt | old | 0.27 | 2.24 | 9.02E-01 | 0.62 | 2.82 | 8.26E-01 | -2.78 | 3.85 | 4.71E-01 |

Genotypic models with major allele homozygous genotype as a reference for all polymorphisms.

AgeNN denotes age cut off separating subsamples of old (age > AgeNN) and young (age <= AgeNN) individuals.

Sample size of the youngest group aged 30 years and younger was 3,068.

Sample size of the oldest group older than 90 years was 1,499.

Nind/Nobs means number of individuals/observations.

Total number of individuals is not equal to that of the sample size because an individual appear in both group if there are measurements at respective ages.
